# Prediction model of axillary lymph node status using an automated breast volume ultrasound radiomics nomogram in early breast cancer with negative axillary ultrasound

**DOI:** 10.3389/fimmu.2025.1460673

**Published:** 2025-03-12

**Authors:** Qianqing Ma, Junli Wang, Zhengzheng Tu, Jingwen She, Jianhui Zhu, Feng Jiang, Chaoxue Zhang

**Affiliations:** ^1^ Department of Ultrasound, The First Affiliated Hospital of Wannan Medical College, Wuhu, China; ^2^ Department of Ultrasound, The First Affiliated Hospital of Anhui Medical University, Hefei, China; ^3^ Department of Ultrasound, The Second People’s Hospital of Wuhu, Wuhu, China; ^4^ Anhui Provincial Key Laboratory of Multimodal Cognitive Computation, School of Computer Science and Technology, Anhui University, Hefei, China; ^5^ Department of Ultrasound, West China Second Hospital, Sichuan University, Chengdu, China

**Keywords:** ultrasound, breast cancer, machine learning, radiomics, nomogram, lymphatic metastasis, automated breast volume ultrasound

## Abstract

**Background:**

Construction and validation of an automated breast volume ultrasound (ABVS)-based nomogram for assessing axillary lymph node (ALNs) metastasis in axillary ultrasound (AUS)-negative early breast cancer.

**Methods:**

A retrospective study of 174 patients with AUS-negative early-stage breast cancer was divided into a training and test with a ratio of 7:3. Radiomics features were extracted by combining images of intra-tumor and peri-tumor ABVS. Select the best classifier from 3 machine learning techniques to build Model 1and radiomics-score (RS). Differences in ER, PR, Her-2, Ki-67 expression were analyzed for intra-tumoral and peri-tumoral habitat radiomics features. Model 2 (based on sonogram features) and Model 3 (based on RS and sonogram features) were constructed by multivariate logistic regression. Efficiency of the models was evaluated by the area under the curve (AUC). Plotting the nomogram and evaluating its treatment in ALN≥3 according to Model 2 and Model 3.

**Result:**

Intratumoral and peritumoral 5 mm radiomics features were screened using least absolute shrinkage and selection operator (LASSO), and logistic regression was used as a classifier to build the best-performing Model 1. Using unsupervised cluster analysis, intratumoral and peritumoral 5mm were classified into 3 habitats, and they differed in PR and Her-2 expression. Model 2 (combining diameter and microcalcification) and Model 3 (combining RS and microcalcification) were created by multivariate logistic regression. Model 3 achieves the highest AUC in both the training (0.827) and validation (0.768) sets. The Nomo-score was calculated based on nomogram-model2 and nomogram-model3, revealing a positive correlation between ALN burden and Nomo-score. Combined with the optimal thresholds, nomogram-model2 screened 54.6%-100% of patients with ALN ≥3 and nomogram-model3 screened 81.8%-100% of patients with ALN ≥3.

**Conclusion:**

The ABVS-based nomogram is an effective tool for assessing ALN metastasis, and it can provide a preoperative basis for individualized treatment of breast cancer.

## Introduction

Axillary lymph node (ALN) status is the most strongly associated factor affecting the survival and prognosis of breast cancer patients (1, 2). Although sentinel lymph node (SLN) biopsy is widely used to detect ALN status. However, the false-negative rate of SLNB is 5.5%-16.7% (3, 4), and SLNB may be associated with sensory nerve damage, lymphedema, and limited shoulder motion (5). In addition, according to the ACOSOG Z0011 trial, patients with SLN metastases of less than 2 early-stage breast cancers who do not undergo axillary surgery do not have affected overall survival and disease-free survival rates (6, 7). Therefore, ALN status is adequately assessed by radiographic methods prior to surgery to minimize unnecessary invasive exploration.

The most valuable radiological method for assessing ALNs is axillary ultrasound (AUS) (8–10). Owing to the noninvasive and inexpensive nature of ultrasound, it is very suitable for long-term observation of ALNs. Approximately 15.5-35.0% of AUS negatives show positivity on pathology, which makes it particularly important to reduce false negatives (11–13). Studies have shown that adding tumor characteristics to the diagnosis of ALNs can significantly reduce this deficiency of AUS (13). Certain ultrasound features of primary breast cancer are associated with ALN metastasis, such as the diameter of the lesion, microcalcification, architectural distortion, and tumor distance from the skin (13–18).

Furthermore, with the advancement of pattern recognition tools, radiomics has attracted increasing interest. Combining the imaging features of a lesion with radiomics features has greatly improved diagnostic performance (17, 19, 20). Radiomics is the process of converting medical images into mineable data by extracting high-throughput quantitative features that effectively reduce inter-examiner variability. In the past, free-form imaging methods and the reliance on adjustable parameters during the examination made the development of radiomics in ultrasound difficult (21), and the emergence of an automatic breast volume scanner (ABVS) has solved this problem. ABVS allows comprehensive and standardized scanning of the entire breast and coronal and sagittal display in specific workstations. The coronal plane of the lesion provides additional information for diagnosis (22), and standardized images also open up new opportunities for ultrasound radiomics (23).

Therefore, we aimed to develop and validate two nomograms (one based on sonogram features and the other based on radiomics and sonogram features) for assessing ALN status in AUS-negative early-stage breast cancers and to explore the value of the nomogram for the preoperative assessment of patients with ALN ≥3.

## Materials and methods

### Patient

The study was approved by the Institutional Review Board and requirement for informed consent was waived (PJ2023-07-11).

A retrospective collection of 499 patients who were diagnosed with breast cancer by histopathology between November 2017 and August 2021 and underwent ABVS at our institution was performed. The inclusion criteria were as follows: (1) patients who underwent SLNB or ALND; (2) unifocal masses ≤ 5 cm in diameter (T1 and T2 stages); (3) ABVS within 2 weeks prior to SLNB or ALND; and (4) no suspicious lymph nodes on AUS (asymmetric or diffuse thickening of the lymph node cortex, an LN with a cortex thickness ≥ 3 mm loss of hilum, and nonhilar blood flow) (24). The exclusion criteria were as follows: (1) patients who received chemotherapy, radiotherapy or endocrine therapy, (2) patients with multifocal or bilateral breast cancer, and (3) patients with nonoccupying lesions without demarcated borders (**Appendix Figure 1**). 174 patients were finally included in this study and randomized into training and test sets at a ratio of 7:3.

Baseline clinical data were acquired from the patient medical record system, including age, pathologic type, immunohistochemical (IHC) results and postoperative ALN status (ALN+ or ALN-). ALN+ patients were further classified into low burden (1-2 ALNs) and high burden (≥3 ALNs) groups. IHC was performed to assess HER-2, Progesterone Receptor (PR), Estrogen Receptor (ER) and Ki-67 status and recorded as positive (+) or negative (-).

### Image acquisition and assessment

Details of image acquisition are provided in **Appendix A.1**. Reader 1 and Reader 2 (11 and 20 years of experience, respectively) analyzed these images. Cases of disagreement were independently resolved by a third Reader (Reader 3, 25 years of experience). None of the sonographers had knowledge of the pathological results. Orientation (parallel or not parallel), margin (circumscribed, noncircumscribed), shape (regular or irregular), echogenicity (hypoechoic, hybrid echo or extremely low echo), microcalcification, tumor location (left, right), quadrant (upper inner, lower inner, upper outer, lower outer, central), convergence sign, distance from nipple, and distance from skin and maximum diameter of the lesion were recorded.

### Segmentation and extraction of radiomics features

**Figure 1:**
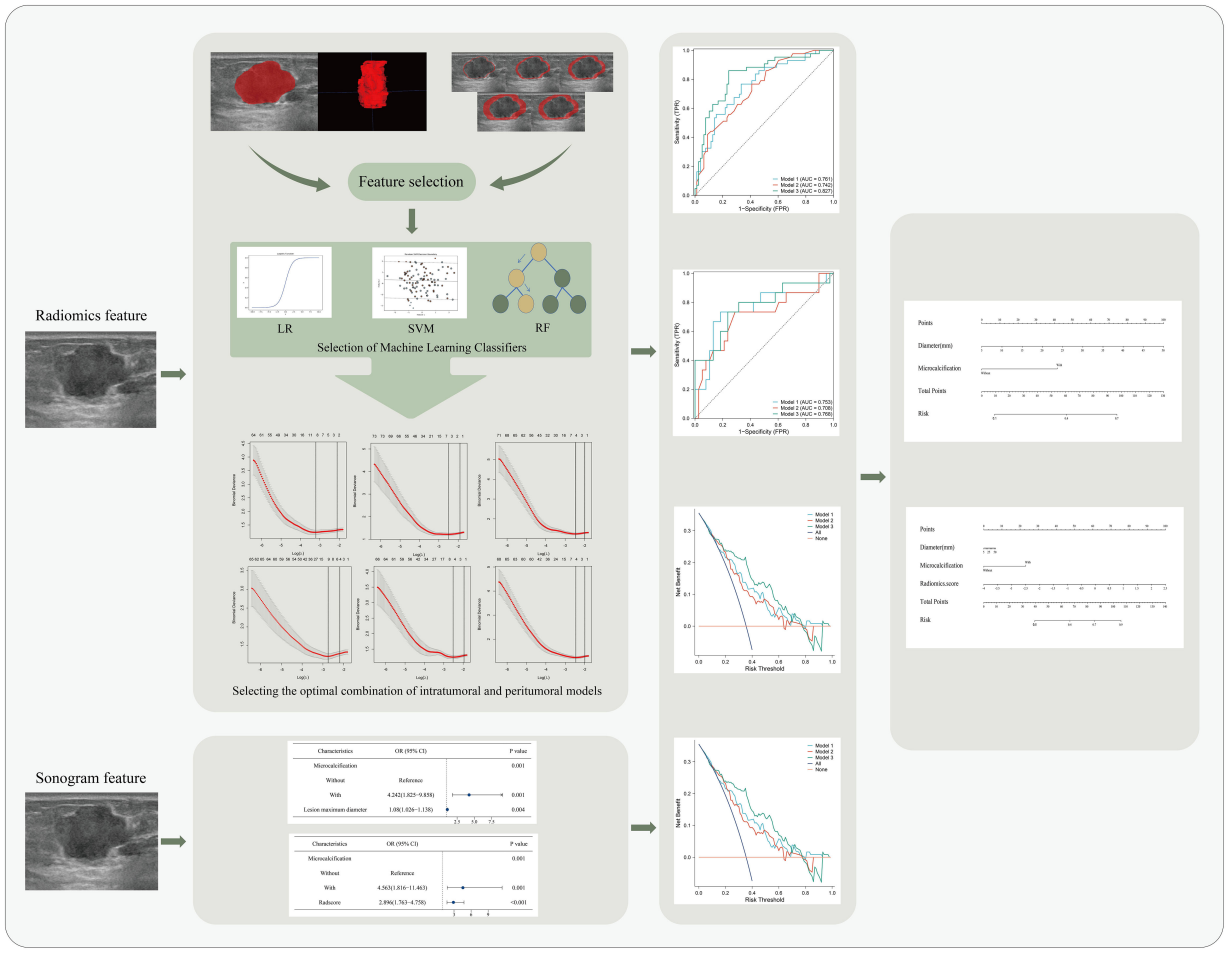
Flowchart of a nomogram for predicting lymph node metastasis in breast cancer.LR, logistic regression; SVM, support vector machine; RF, random forest; OR, odds ratio; CI, confidence interval.

**Figure 2:**
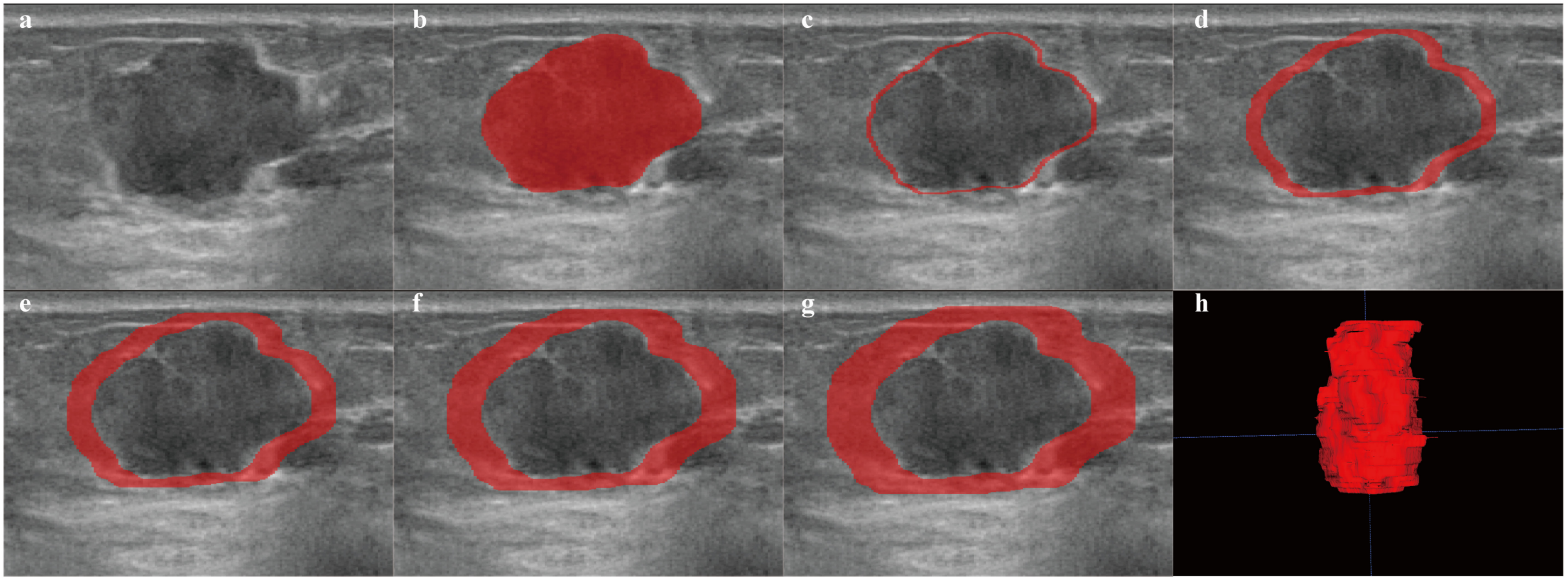
Examples of ROI segmentation on ABVS. The red area represents the ROI of the lesion. **(a)** image of the lesion. **(b–g)** ROIs for regions of 1 mm, 3 mm, 5 mm, 7 mm, and 9 mm around the tumor, respectively. **(h)** 3D image of the intratumoral region of the lesion.

The flowchart of the study was showed in **Figure 1**. Tumor regions of interest (ROIs) were manually depicted slice-by-slice by Reader 1 using ITK-SNAP 3.6. Next, using Python 3.7, areas 1, 3, 5, 7, and 9 mm around the ROI will be generated. **Figure 2** shows an example of the ROI segmentation and expansion process for ABVS. Fifty images were randomly selected and re-segmented by Reader 1 and Reader 2 after 2 weeks to calculate intra- and inter-class correlation coefficients (ICC). Radiomics features were extracted using the “PyRadiomics (3.7.0)” Python package (Detailed in **Appendix A.2**).

### Feature screening, machine learning classifier, and Model 1 building

The obtained intratumoral region (ITR) features are preprocessed and the model is constructed using classifiers employing logistic regression (LR), random forest (RF) and support vector machine (SVM). An optimal ML classifier was used to construct a joint ITR and peritumoral region (PTR) radiomics model, named Model 1. The radiomics-score (RS) was calculated for each patient. Methods of preprocessing and ML classifiers are shown in the **Appendix A.3**.

### Radiomics habitat analysis

The k-means method performed cluster analysis on subregions using all data without splitting into training and testing sets. The optimal number of clusters was determined by the elbow method after plotting the within-cluster sum of squares (WCSS) with k values (25). The number of clusters from 1 to 10 was tested in this study. The ITR and PTRs were divided into subregions (habitats) based on optimal k values. Extraction of valuable radiomics features in each habitat and analysis of these features in relation to ER, PR, HER-2, and Ki-67 were conducted.

### Construction of Model 2 and Model 3

For sonographic feature variables, univariate logistic regression was used to evaluate their correlation with lymph node metastasis, and factors with *P*<0.1 were introduced into multivariate logistic regression. Factors with *P*<0.05 were identified as independent predictors, whereby an ultrasound feature model was created and named Model 2. RS was combined with ultrasound features to create a sonography radiomics model named Model 3. The Nomo-score was determined for each patient according to Model 2 and Model 3.

### Assessment of the models and construction of the nomogram

The predictive performance of Model 1, Model 2, and Model 3 was evaluated by area under curve (AUC) of receiver operating characteristic (ROC), accuracy, sensitivity, and specificity. Plotting Decision Curve Analysis (DCA) assesses the net benefits of the model. Finally, nomogram-model 2 and nomogram-model 3 were constructed based on Model 2 and Model 3.

Associations between Nomo-scores and ALN burden were assessed using correlation analysis. Furthermore, the value of the model for ALN ≥ 3 was assessed based on the Nomo-score cutoff value. (Statistical analyses, **Appendix A.4**.)

## Results

### Patient characteristics

The 174 patients enrolled in this study were randomized into a training set (n=121) and a test set (n=53) at a ratio of 7:3. **Table 1** displays the distribution of the patients, and no significant difference in training and test sets.

**Table 1:** The distribution of the patients.

Variables		Training set	Test set	*P*
Age (y)		51.9 ± 12.02	53.87 ± 11.77	0.319
ALN	ALN-	78 (64.5%)	38 (71.7%)	0.351
	ALN+	43 (35.5%)	15 (28.3%)	
Histological type	Invasive ductal carcinoma	96 (79.3%)	38 (71.7%)	0.508
	Invasive lobular carcinoma	8 (6.6%)	4 (7.5%)	
	Others	17 (14%)	11 (20.8%)	
Estrogen receptor	Negative	30 (24.8%)	15 (28.3%)	0.627
	Positive	91 (75.2%)	38 (71.7%)	
Progesterone receptor	Negative	28 (23.1%)	10 (18.9%)	0.53
	Positive	93 (76.9%)	43 (81.1%)	
HER-2	Negative	70 (57.9%)	34 (64.2%)	0.652
	Positive	26 (21.5%)	11 (20.8%)	
	Missing	25 (20.7%)	8 (15.1%)	
Ki-67	Negative (<14%)	26 (21.5%)	15 (28.3%)	0.33
	Positive (>14%)	95 (78.5%)	38 (71.7%)	
Lesion maximum diameter (mm)		22.26 ± 8.17	21.76 ± 10.06	0.731
Distance from nipple (mm)		41.26 ± 21.24	40.02 ± 20.26	0.719
Distance from skin (mm)		6.78 ± 2.9	7.35 ± 3.78	0.283
Tumor location	Left	66 (54.5%)	29 (54.7%)	0.983
	Right	55 (45.5%)	24 (45.3%)	
Quadrant	Upper inner	33 (27.3%)	6 (11.3%)	0.109*
	Lower inner	13 (10.7%)	8 (15.1%)	
	Upper outer	47 (38.8%)	22 (41.5%)	
	Lower outer	27 (22.3%)	15 (28.3%)	
	Central	1 (0.8%)	2 (3.8%)	
Shape	Regular	19 (15.7%)	7 (13.2%)	0.671
	Irregular	102 (84.3%)	46 (86.8%)	
Margin	Circumscribed	12 (9.9%)	7 (13.2%)	0.522
	Non-circumscribed	109 (90.1%)	46 (86.8%)	
Orientation	Parallel	95 (78.5%)	42 (79.2%)	0.913
	Non-parallel	26 (21.5%)	11 (20.8%)	
Microcalcification	Without	80 (66.1%)	36 (67.9%)	0.816
	With	41 (33.9%)	17 (32.1%)	
Echogenicity	Hypo-echoic	105 (86.8%)	48 (90.6%)	0.176
	hybrid echo	3 (2.5%)	3 (5.7%)	
	Extremely low echo	13 (10.7%)	2 (3.8%)	
Convergence sign	No	42 (34.7%)	18 (34%)	0.924
	Yes	79 (65.3%)	35 (66%)	

ALN, axillary lymph node; HER-2, human epidermal growth factor receptor 2 ^*^The likelihood ratio test is used.

### Feature selection, classifiers performance comparison and Model 1 construction

A total of 1688 features were extracted from the ITR and models were constructed using LR, SVM and RF classifiers. The AUC of the LR classifier is highest in the training (0.718) and test (0.725) sets. **Appendix A.5** and **Appendix Table 1** provide the data preprocessing procedure, three classifier structures and performance.

Extracted features of PTR_1mm_, PTR_3mm_, PTR_5mm_, PTR_7mm_ and PTR_9mm_ PTR. The ITR is modeled in conjunction with the PTR using the LR classifier. ITR+PTR5mm model had AUCs of 0.761 and 0.753 in the training and test sets, respectively, and showed balanced sensitivity, specificity, and accuracy. It was used as the final radiomics model, named Model 1. Based on this model, RS was calculated for each patient. The feature screening and building methods are described in **Appendix A.6** and **Appendix Table 2**. The performance of the radiomics models for the ITR and PTR combinations are shown in **Table 2**.

**Table 2:** The performance of the radiomics model for the intratumor and peritumor combinations.

	Train				Test			
	AUC (95%CI)	ACC	SEN	SPE	AUC (95%CI)	ACC	SEN	SPE
ITR	0.718 (0.626-0.81)	0.661	**0.814**	0.577	0.725 (0.56-0.889)	0.774	**0.733**	0.789
ITR+ PTR_1mm_	0.734 (0.639-0.829)	0.669	0.744	0.628	**0.774** **(0.611-0.936)**	**0.811**	0.667	**0.868**
ITR+ PTR_3mm_	0.725 (0.627-0.824)	**0.760**	0.442	**0.936**	0.732 (0.569-0.894)	0.774	0.667	0.816
ITR+ PTR_5mm_	**0.761** **(0.673-0.85)**	0.702	0.767	0.667	0.753 (0.584-0.921)	0.792	**0.733**	0.816
ITR+ PTR_7mm_	0.732 (0.636-0.828)	0.694	0.721	0.679	0.718 (0.555-0.88)	0.736	0.600	0.789
ITR+ PTR_9mm_	0.735 (0.64-0.83)	0.702	0.721	0.692	0.712 (0.55-0.875)	0.736	0.600	0.789

Bold characters in the table indicate the best performance for each metric in this study. ITR, intratumoral region; PTR, peritumoral region; ACC, accuracy; SEN sensitivity; SPE, specificity.

### Radiomics habitat analysis

**Figure 3:**
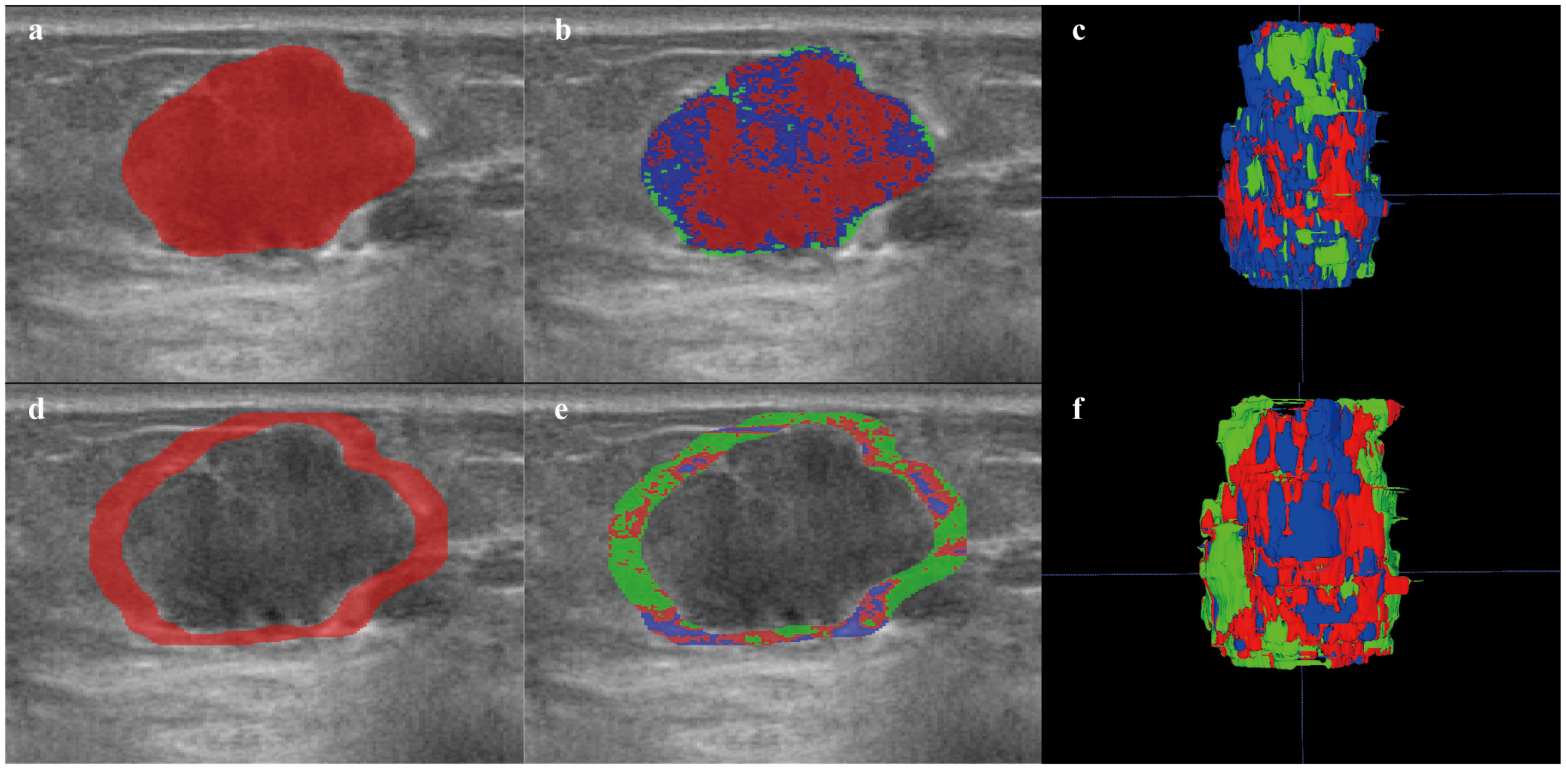
Habitat image of breast lesions ITR and PTR_5mm_. **(a)** ROI of ITR. **(b)** habitat image of the ITR. **(c)** three-dimensional imaging of ITR habitat image. **(d)** ROI of PTR_5mm_. **(e)** habitat image of the PTR_5mm_. **(f)** three-dimensional imaging of the PTR_5mm_ habitat image. Red, green, and blue represent Habitat-1, Habitat-2, and Habitat-3, respectively. ITR, intratumoral region; PTR, peritumoral region.

Following the k-means clustering method, ITR and PTR_5mm_ were classified into three habitats (**Appendix Figure 2**, **Figure 3**). Detailed steps for the habitat analysis are given in **Appendix A.7**. The results show that the exponential_ngtdm_Busyness of PTR_5mm_ Habitat-2 differed in PR expression (*P*=0.034) and Exponential_ngtdm_Busyness of PTR_5mm_ Habitat-3 differed differs in HER-2 expression (*P*=0.004). Habitat radiomics features with the distribution of ER, PR, HER-2, and Ki-67 expression are shown in the **Appendix Table 3**.

### Model 2 and Model 3 establishment

Multivariate regression in the training set showed that diameter and microcalcification were independent predictors of ALN+ (**Table 3**). These factors were used to construct Model 2. After adding RS features to the model, RS and microcalcification were independent predictors of ALN+, and these factors were used to construct Model 3. The Nomo-score of the patients was calculated as follows:

**Table 3:** Univariate and multivariate logistic regression analysis of variables associated with ALN+.

	Univariate		Multivariate Model 2		Multivariate Model 3	
	OR (95%CI)	*P*	OR (95%CI)	*P*	OR (95%CI)	*P*
Age	1.012 (0.981-1.044)	0.445	–	–	–	–
Lesion maximum diameter(mm)	1.073 (1.022-1.127)	0.005	1.08 (1.026-1.138)	0.004	–	0.769
Distance from nipple (mm)	0.993 (0.975-1.011)	0.429	–	–	–	–
Distance from skin (mm)	0.989 (0.869-1.126)	0.872	–	–	–	–
Tumor location (Ref. center)	0.798(0.376-1.691)	0.556	–	–	–	–
Quadrant						
Upper inner	Ref.	0.998	–	–	–	–
Lower inner	1.094 (0.291-4.109)	0.894	–	–	–	–
Upper outer	0.903 (0.356-2.292)	0.830	–	–	–	–
Lower outer	1.029 (0.358-2.957)	0.957	–	–	–	–
Central	0	1.000	–	–	–	–
Shape (Ref.Regular)	1.662 (0.555-4.98)	0.364	–	–	–	–
Margin (Ref.Circumscribed)	0.749 (0.223-2.521)	0.641	–	–	–	–
Orientation (Ref.Parallel)	0.95 (0.382-2.361)	0.912	–	–	–	–
Microcalcification (Ref.without)	3.833 (1.726-8.513)	0.001	4.242 (1.825-9.858)	0.001	4.563 (1.816-11.463)	0.001
echogenicity						
Hypo-echoic	Ref.	0.541	–	–	–	–
hybrid echo	3.833 (0.336-43.719)	0.279	–	–	–	–
Extremely low echo	1.198 (0.365-3.929)	0.766	–	–	–	–
convergence sign (Ref.no)	0.988 (0.452-2.159)	0.976	–	–	–	–
RS	2.718 (1.719-4.299)	0.000	–	–	2.896 (1.763-4.758)	<0.001

Ref, reference; RS, radiomics-score; OR, Odds ratio;CI, confidence interval.

Nomo-score (Model 2) =-2.904 + 1.445* Microcalcification  + 0.077*Diameter

Nomo-score (Model 3) =-0.521 + 1.063*RS  +1.518*Microcalcification

**Figure 4:**
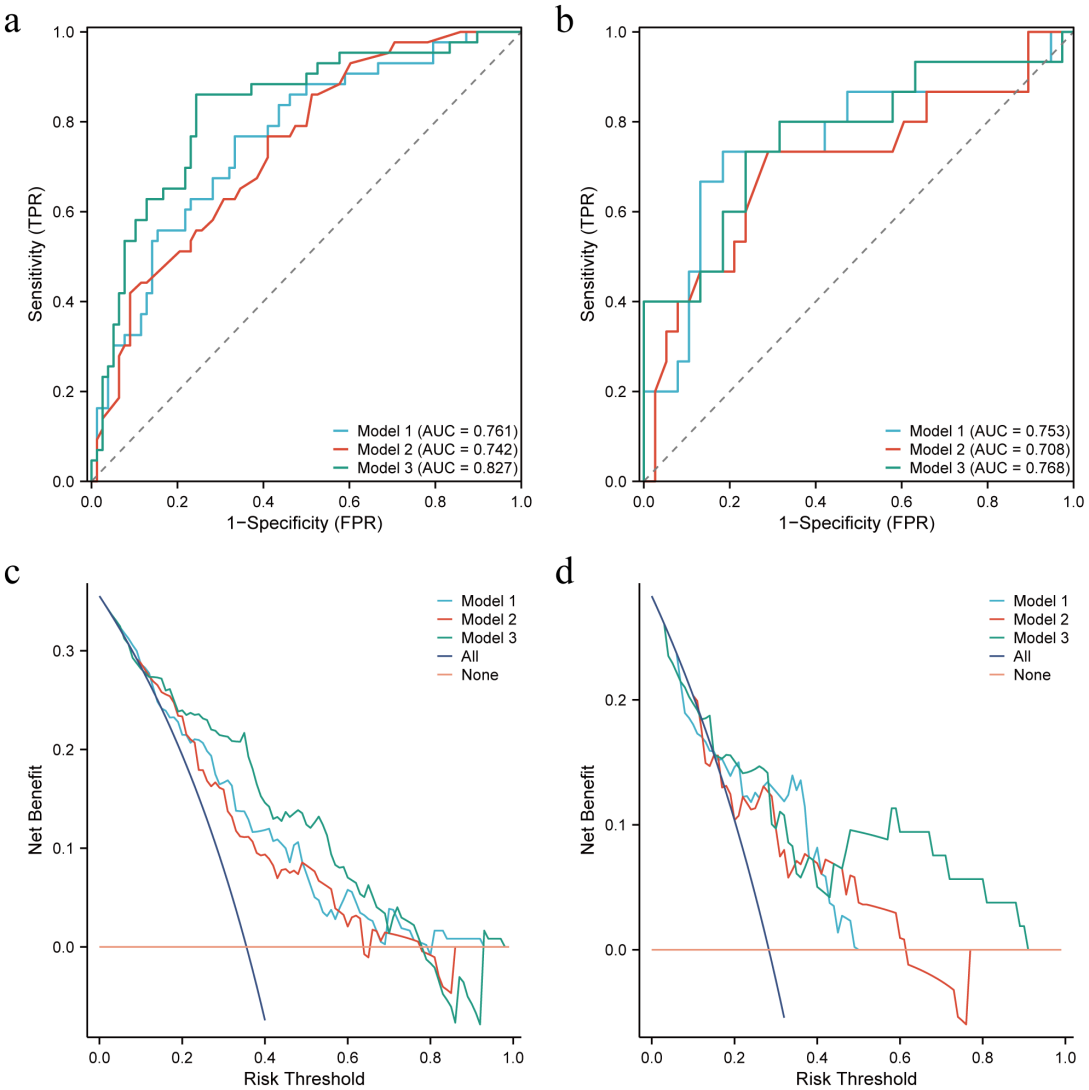
Receiver operating characteristic (ROC) curves and decision curves (DCA) for the three models in the training and test sets. **(a, b)** ROC for the training and test sets of the three models, respectively. **(c, d)** DCA for the training and test sets of the three models, respectively.

The performance of Model 1, Model 2, and Model 3 are shown in **Table 4**. In the training set, model 3 outperforms model 1 and model 2 (Delong test, *P*=0.033 and 0.027, respectively). AUC of Model 3 was also higher than that of the other 2 models in the test set. The ROC curves of the three models are shown in **Figures 4a, b**. The DCA curve also reveals that Model 3 had the highest net benefit in most of the threshold probabilities (**Figures 4c, d**).

**Table 4:** Performance of Model 1, Model 2 and Model 3 in the training and test sets.

	AUC (95%CI)	ACC	SEN	SPE	*P values*
Training					
Model 1	0.761 (0.673-0.85)	0.702	0.767	0.667	0.033
Model 2	0.742 (0.653-0.831)	0.653	0.767	0.59	0.027
Model 3	0.827 (0.748-0.906)	0.793	0.86	0.756	Ref.
Test					
Model 1	0.753 (0.584-0.921)	0.792	0.733	0.816	0.719
Model 2	0.708 (0.534-0.881)	0.717	0.733	0.711	0.352
Model 3	0.768 (0.61-0.927)	0.755	0.733	0.763	Ref.

Ref, reference; *P values* were derived from the DeLong test; CI, confidence interval; ACC, accuracy; SEN, sensitivity; SPE, specificity.

### Assessment of the models and construction of the nomogram

**Figure 5:**
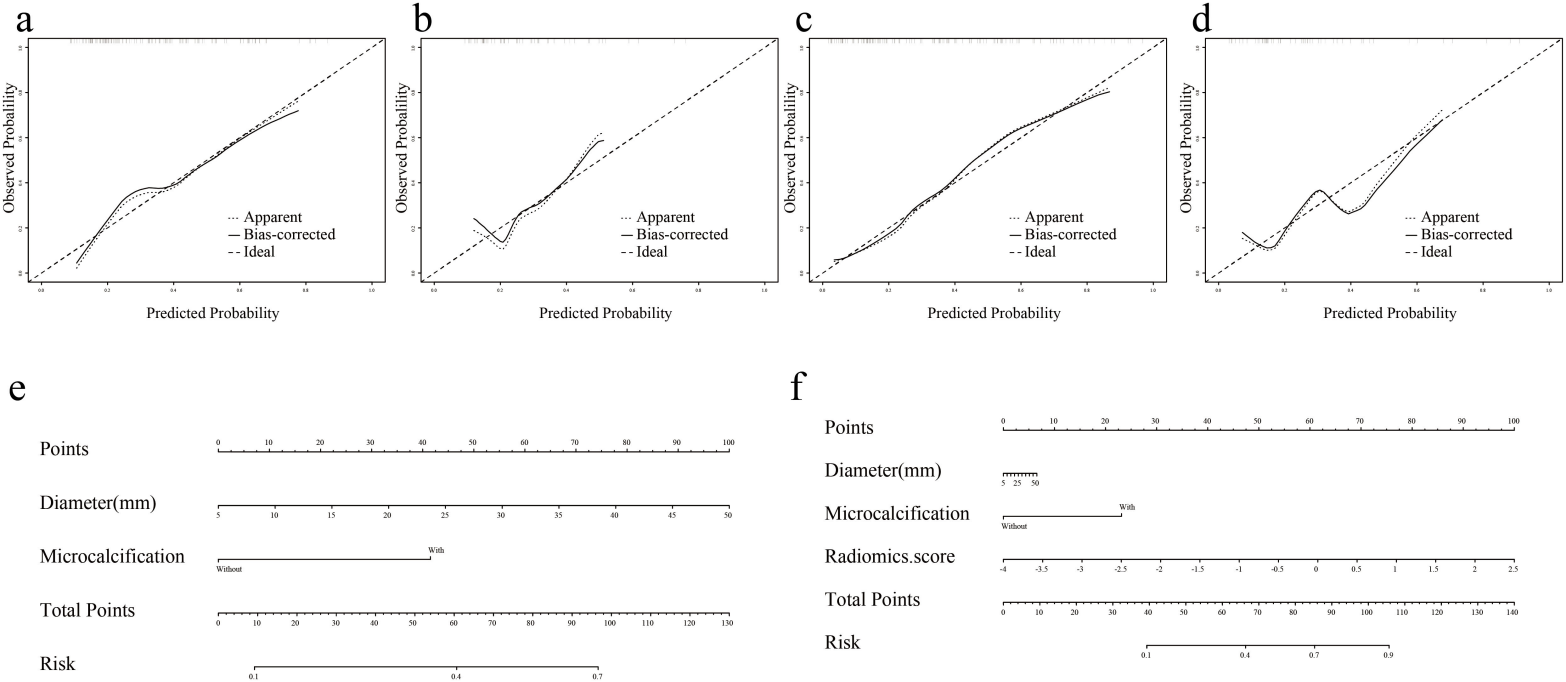
Nomogram and calibration curves of Model 2 and Model 3. **(a, b)** Nomogram-model 2 and nomogram-model 3. **(c, d)** Calibration curves for Nomogram-model 2 in training and test sets. **(e, f)** Calibration curves for Nomogram-model 3 in training and test sets.

Nomogram-model 2 and nomogram-model 3 were constructed based on the factors of Model 2 and Model 3, respectively (**Figures 5a, b**). The calibration curves and nonsignificant Hosmer-Lemeshow test statistics (0.894 and 0.568 for the nomogram-model 2 training and test sets, respectively, and 0.538 and 0.148 for the nomogram-model 3 training and test sets, respectively) show that the training and test sets are well calibrated, suggesting that the nomogram can show good agreement between predictions and observations (**Figures 5c–f**).

**Figure 6:**
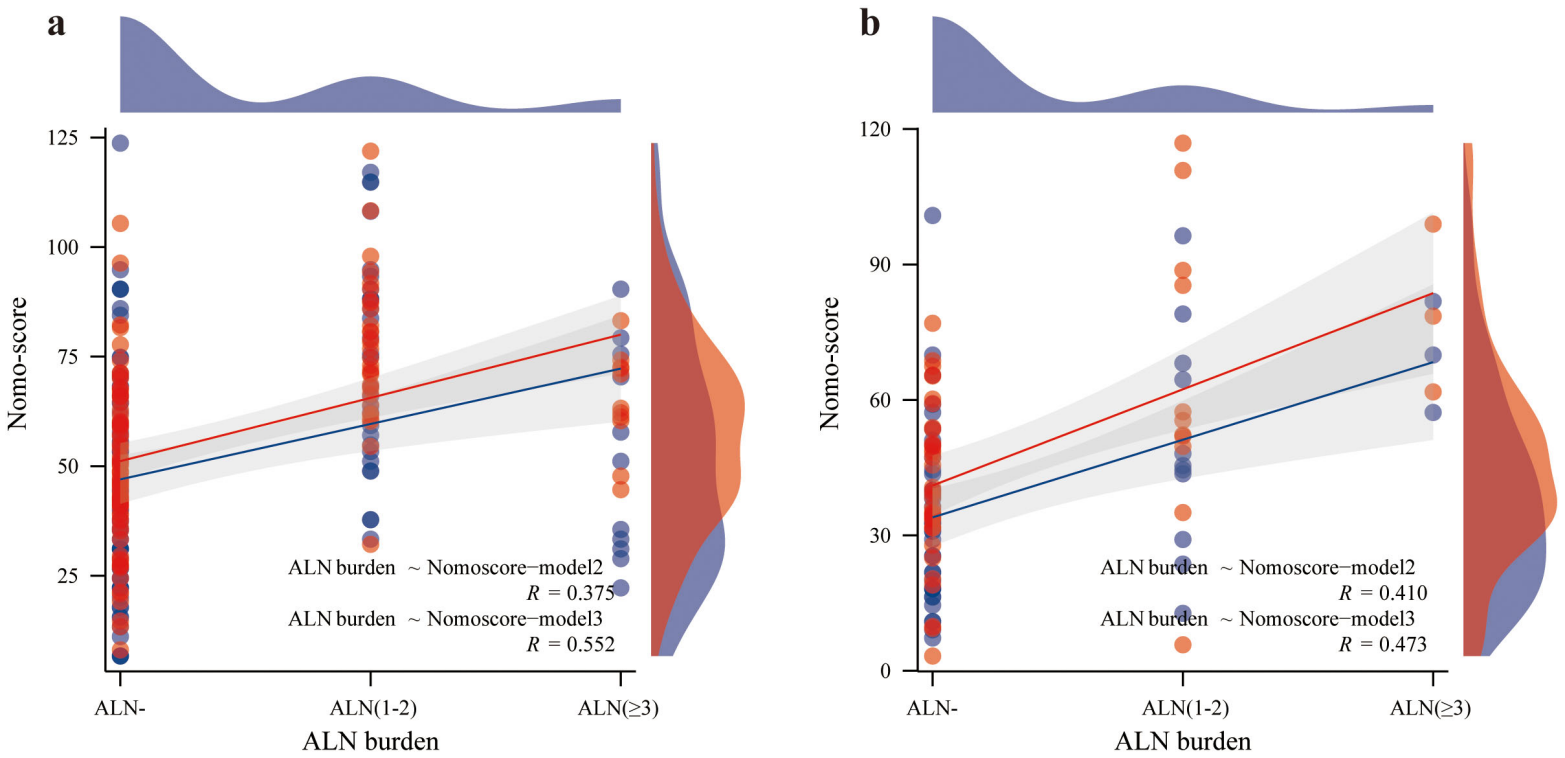
Correlation between ALN burden and Nomo-score. The red line represents the Nomo-score (Model 2), and the blue line represents the Nomo-score (Model 3); Spearman’s correlation coefficient suggests that the higher the ALN burden is, the higher the Nomo score. **(a)** correlation between ALN burden and Nomo-score in the training set. **(b)** correlation between ALN burden and Nomo-score in the test set.

As shown in **Figure 6a**, the Nomo-score (Model 2) was positively correlated with the ALN burden (training set *r*=0.375, *P*<0.001; test set *r*=0.410, *P*=0.002). The optimal threshold when using the Nomo-score to predict ALN state was 47.778 (Youden=0.357). When the Nomo-score was ≥47.778, 54.6% (6/11, training set) and 100% (3/3, test set) of patients had at least 3 ALNs.

As shown in **Figure 6b**, the Nomo-score (Model 3) was also positively correlated with the ALN burden (training set *r*=0.552, *P*<0.001; test set *r*=0.473, *P*<0.000). The optimal threshold when using the Nomo-score to predict ALN state was 60.237 (Youden=0.616). When the Nomo-score was ≥60.237, 81.8% (9/11, training set) and 100% (3/3, test set) of patients had at least 3 ALNs.

## Discussion

In this study, we developed nomogram-model 2 and nomogram-model 3 capable of effectively assessing ALN metastasis in AUS-negative early breast cancer with AUC, accuracy, sensitivity, and specificity of 0.708, 0.717,0.733, 0.711; 0.768, 0.755, 0.733, 0.763, respectively. This method was effective in reducing unnecessary SLNB. Both nomograms are also effective in assessing axillary lymph node burden, and it helps clinics make effective treatment decisions, thereby reducing over- or under-treatment. In addition, habitat analysis showed that the radiomics features of the PTR_5mm_ subregion differed in the expression of PR and HER-2, which provided a basis for the validity of the model.

Axillary staging, in addition to biological and anatomical staging of the tumor, is very important in the treatment and prognosis of breast cancer. AUS is the most effective radiologic method for evaluating ALNs (8–10). Ultrasound reporting of suspicious LNs has been shown in several studies to have a significant influence in predicting ALN+ (12, 13, 26), but ultrasound has a high false-negative rate of predicting ALNs (15.5%-35.0%) (11–13). To maximize the performance of ultrasound in predicting ALNs, we focused on ultrasound-negative ALNs.

In the construction of Model 1, we differ from other models that only analyze the radiomics features within the tumor (12); we combined ITR and PTR features and obtained the best imaging histology model in the ITR+PTR_5mm_ model. Three radiomics features were screened, one from the ITR (wavelet.LLL_glcm_Imc2) and two from the PTR (exponential_ngtdm_Busyness, wavelet.LHL_glszm_SizeZoneNonUniformityNo- rmalized). This suggests that the PTR improves predictive performance in radiographic analysis (27). This is because the peritumoral environment secretes many growth factors and cytokines that can induce hypoxia and angiogenesis, which play an important role in tumorigenesis, progression, or metastasis. Integrating tumor and peritumor data allows for a more comprehensive characterization of tumor invasion and metastasis (28).

The complex vascular system within a tumor result in intratumoral heterogeneity. Heterogeneity is uniformly scattered across the tumor, and regional phenotypic variation inside the tumor is then overlooked (29). Based on speculation by some scholars, the subregions containing voxels with similar imaging characteristics will share common tumor biology properties (30, 31). Habitat analysis methods were developed to divide tumors into subregions containing clusters of voxels with similar characteristics, which allows for better quantification of heterogeneity within the tumor (32, 33). Recently the method has made significant breakthroughs in the assessment of lung, colorectal, and ovarian cancers (34, 35). In this study, an unsupervised clustering method was used to classify the ITR and the PTR_5mm_ area of breast tumors into three categories. The results showed that the distribution of the exponential_ngtdm_Busyness feature of Habitat-2 and the exponential_ngtdm_Busyness feature of Habitat-3 in the PTR_5mm_ was correlated with the expression of PR and HER-2, and it has been demonstrated that patients with different expression of PR and HER-2 tended to have differences in ALN status (12, 36, 37). This provides strong evidential support for our model and further demonstrates the reliability of the model in predicting ALN status in breast tumors.

Ultrasound characteristics of the tumor are also valuable in the diagnosis of ALN metastases. In a recent report, Xiong et al. (18) combined sonographic features and clinicopathologic features for the prediction of AUS-negative ALNs, with AUCs of 0.705 and 0.745 for the training and test sets, respectively, and showed results similar to those of model 2 in this study. However, pathology indicators were not included in our nomogram, and some histopathology can only be assessed after surgical resection or after aspiration, which may limit the use of the model. Furthermore, in our study, microcalcification was an important factor in the diagnosis of ALN+ (13). Calcification of the tumor is often indicative of a poor prognosis (38), and calcium deposits in the necrotic areas of the tumor appear as gravel or pinpoints, occurring mainly in clusters. Xiong et al. (18) did not analyze calcification, probably because ultrasound does not preserve all images of the lesion, making it difficult to perform an accurate analysis in a retrospective study of the presence or absence of calcification in the lesion. With ABVS, in addition to preserving the complete breast image for subsequent analysis, it also shows microcalcifications more clearly than conventional ultrasound (39). Irregularly shaped ultrasound features in breast cancer significantly correlate with ALN metastasis (40). In invasive breast cancer, cancer cells may infiltrate the surrounding tissue at varying growth rates, creating inconsistent tumor margins. The margins may produce irregular shapes, whereas margins presenting as fuzzy, microfollicular, or acinar may not directly form irregular shapes (13). In our study, however, there was no significant difference in AUS-negative lesions, but the availability of peripheral regions in radiomics explains this phenomenon. Differences in the margins of AUS-negative tumors have altered textural features, although they cannot be recognized by the naked eye. The diameter of the lesion is a major predictor of ALN as well (13, 15–17). Larger breast cancers have more extensive glandular invasion by cancer cells and a higher likelihood of metastatic ALNs via lymphatic drainage (41). The results of Model 2 also showed that tumor diameter was an important predictor of ALN metastasis, which is consistent with previous reports. In patients with invasive breast cancer, there is a nonlinear association between tumor size and ALN metastasis (13, 16), which further emphasizes the important role of tumor diameter in predicting ALN metastasis. Sonogram features are usually combined with radiomics features to further improve diagnostic performance (17, 19, 20). In this study, Model 3 combines the features of Model 1 and Model 2, and it obtained the highest AUC (training set: 0.827, test set: 0.768), and the DCA curve also showed that model 3 had a high net benefit.

Based on the results of the Z0011 trial, the clinical practice guidelines were updated to state that women with T1 or T2 primary invasive breast cancer with 1-2 metastatic SLNs who are scheduled to undergo breast-conserving surgery with whole-breast radiotherapy no longer need ALND (6, 42). Assessing ALN burden is also critical. The nomogram in this study was able to differentiate between ALN- and ALN+ patients. It also has better results for identifying ALN statuses with a high burden. A positive correlation between the Nomo-score and ALN burden is shown in **Figure 6**. The higher the Nomo-score is, the greater the likelihood of a high ALN burden. Combined with the optimal thresholds, nomogram-model 2 screens 54.6-100% of those with an ALN high burden. Nomogram-model 3 screens 81.8-100% of those with a high ALN burden. This will increase surgeon confidence in performing ALND based on a positive SLN.

There are some limitations in this study. First, this was a single-center retrospective study. The nongeneralizability of the ABVS examination resulted in a small sample size for this study, and prospective and multicenter studies may be conducted in the future for further validation. Second, influenced by different regional breast cancer management guidelines and subjective patient factors, some HER-2 positive patients in this study did not receive neoadjuvant therapy prior to surgery. In future studies, we will further expand the sample size and include patients receiving neoadjuvant therapy, so as to provide treatment recommendations for more breast cancer patients. Third, the ROI for each lesion was manually defined, and although we removed features with ICCs < 0.75, interobserver variability was unavoidable. In the future, we will solve this by automatic or semiautomatic segmentation. Lastly, owing to the difficulty of accurately drawing ROIs for nonoccupying lesions without borders and the difficulty of identifying which lesion is the cause of metastatic ALN for multiple lesions, these two types of lesions were excluded. This may cause selection bias and nomogram restriction.

In conclusion, two nomograms were developed and validated in this study: one based on sonogram features named nomograms-Model 2; and one based on sonogram and radiomics features named nomograms-Model 3. They can predict ALN status more accurately in AUS-negative breast cancer patients and also show good performance in assessing ALN burden. These two nomograms can effectively help clinicians in the preoperative assessment of ALN status and contribute to the optimization of clinical decision-making in breast cancer patients.

## Data Availability

The raw data supporting the conclusions of this article will be made available by the authors, without undue reservation.
