# Supplementary material for: Prediction model of axillary lymph node status using an automated breast volume ultrasound radiomics nomogram in early breast cancer with negative axillary ultrasound
Source: Front Immunol. 2025 Mar 12;16:1460673. doi: 10.3389/fimmu.2025.1460673 (PMC11937125; doi:10.3389/fimmu.2025.1460673)
Supplement: Supplementary file 1 [file Presentation1.pdf]

Prediction Model of Axillary Lymph Node Status Using an Automated Breast Volume Ultrasound (ABVS) Radiomics Nomogram in Early Breast Cancer with Negative Axillary Ultrasound Lymph Nodes

Appendix A.1

Image acquisition

Image acquisition was performed by a radiologist (7 years of experience) using an ACUSON S2000 Automated Breast Volume Scanner (Siemens Medical Solutions, Inc., Mountain View, CA, USA). A 15 cm wide linear array transducer (5-14 MHz) was first used to perform a continuous, automated sweep of the breast in the median and lateral positions, with additional planes scanned as necessary. Breast and axilla were then scanned using a 9L transducer (7-14 MHz). All images were saved in DICOM format in the workstation.

Appendix A.2

Extraction of image features

The radiomics features were extracted using the "pyradiomics" package in Python (version 3.7). In addition to the original image, we also extracted features from the wavelet, LoG, square, square root, logarithm, exponential, gradient, LBP (local binary pattern)2D, and LBP3D. In total, 1688 features were extracted from each image. There were 14 shape features, 324 first order features, 432 gray level cooccurrence

matrix (GLCM) features, 252 gray level dependence matrix (GLDM) features, 288 gray level run length matrix (GLRLM) features, 288 gray level size zone matrix (GLSZM) features, and 90 neighboring gray tone difference matrix (NGTDM) features.

Detailed descriptions of feature extraction methods, image types and parameter settings are provided in the radiomics documentation. (https://pyradiomics.readthedocs.io/en/latest).

Appendix A.3

Methods of preprocessing and machine learning classifiers

First, intra- and interobserver variability was investigated, and radiomics features with ICC > 0.75 were removed. Then, the feature distributions of all the data were normalized using the Z-score transform. In addition, we removed features in the training set with Pearson correlation coefficients > 0.8 to minimize the validity covariance of the variables. The model was then built using classifiers employing logistic regression (LR), random forest (RF) and support vector machine (SVM) machine learning(1-3).

LR: A linear model widely used for binary classification problems that outputs the probability value of a sample belonging to a positive class, allowing an intuitive understanding of the likelihood of a sample belonging to a positive class. In this study, in order to minimize model overfitting or selection bias, the least absolute shrinkage and selection operator (LASSO) with 10-fold cross-test was used to select the most

salient features suggestive of ALN states. Finally, LR was used to build the best predictive model.

RF: The performance and generalization ability of the model is improved by constructing multiple decision trees and taking the average or majority vote of their results, which effectively reduces the risk of overfitting and is suitable for classification problems that require high accuracy. In this study mtry is set as the square root of the total variable and the ntree corresponding to when the error is lowest is found.

SVM: In classification problems, the goal of SVM is to find a hyperplane that divides the dataset into two classes and maximizes the distance from the hyperplane to the nearest data point (support vector). It is suitable for datasets that are relatively small and have high feature dimensionality. Modeling is performed using linear kernel function, polynomial kernel function, radial basis kernel function and sigmoid kernel function and the best method is selected.

Appendix A.4

Statistical Analysis

All statistical analyses were performed using R software 4.1.2 and SPSS (version 25.0). Continuous data were reported as the mean ± standard deviation or median (interquartile range). Categorical variables were compared between the training set and test set using the $\chi 2$ test, fisher test, or likelihood ratio test and continuous variables were compared using Mann-Whitney *U* test or Student's *t*- test. The Pearson

or Spearman rank correlation test was used to assess the correlation of Nomo-score and ALN state. All the levels of statistical significance were two-sided, and *P values* < 0.05 were considered significant.

The “irr” package was used for the intra-inter class correlation coefficient. The "caret" package was used to calculate the Z score of the data as follows: $z = (x-\mu)/\sigma$ (x: sample value; μ: mean; σ: standard deviation). The “glm” package was used for multivariate logistic regression analysis. The “glmnet” package was used for LASSO logistic regression. The “rms” package was used to draw the nomogram. The “pROC” package was used to plot the ROC curves and measure the AUCs and perform the DeLong test. The “rms” package was used to plot the calibration curves. The “rmda” package was used to perform DCA. The "randomForest" package was used for RandomForest. The “e1071” package was used for SVM. The Python libraries Scikit-learn was used for k-means clustering.

Appendix A.5

Details of the data preprocessing and the construction of the three classifiers

A total of 1688 features were extracted from the tumor region of the ABVS image, and 1335 features remained after removing the features with ICC<0.75. After removing further correlation features greater than 0.8, we had 139 features remaining. These features were used to construct LR, SVM and RF classifiers.

LR: Further feature screening using least absolute shrinkage and selection operator (LASSO). The 1- standard error of the minimum standard (1-SE standard) is

used to adjust the regularization parameter (λ) and feature selection using tenfold cross-validation. Finally, the model was fitted using LR.

RF: mtry was set to the square root of the total variable (mtry=12). The OOB estimate of the error rate was minimized (37.19%) when ntree was 125.

SVM: Control parameters within 2-9 using recursive feature elimination. The results showed the highest accuracy with 4 variables, and then the model was fitted to the 4 variables using a polynomial kernel function.

The AUC of the LR classifier is highest in the training and test sets. DeLong test shows that the LR classifier outperforms the RF classifier in the training set (P=0.001), and it outperforms the SVM model in the test set (P=0.039). LR classifiers were selected for the next step of model building for the radiomics model.

Appendix A.6

Intratumoral and peritumoral radiomics modeling

1688 features were extracted from the peritumoral region $(PTR)_{1mm}$, PTR_{3mm}, PTR_{5mm}, PTR_{7mm}, and PTR_{9mm}, respectively. After deleting the features with ICC < 0.75, the correlation test was performed by combining the ABVS features with the peritumor features and deleting the features with a Pearson correlation coefficient correlation > 0.8. The remaining features were subjected to LASSO for further screening. The penalty coefficient Log(λ) in the LASSO model was selected by 10-fold cross-validation based on the minimum criterion. The value of λ with the smallest

cross-validation error was chosen as the optimal value of the model. Finally, the modeling was performed using LR. The parameters and performance of the intra-tumor and peri-tumor radiomics models are shown in Appendix Table 3 and Table 1, respectively.

In the training set, the intratumoral region (ITR) model had the highest sensitivity, yet its specificity and accuracy were lower. The ITR+PTR_{3mm} model had higher specificity and accuracy, which was low in sensitivity. In the test set, the ITR+PTR_{1mm} model had high specificity and accuracy but low sensitivity and low accuracy in its training set. Considering the performance of the predictive model in the training and test sets, it was decided that the ITR+PTR_{5mm} model would be used as the final radiomics model; it was named Model 1, and the RS for each patient was calculated based on this model.

Appendix A.7.

Detailed steps for the habitat analysis

ITR and PTR_{5mm} were clustered using the k-means method. The elbow criterion requires finding the "elbow" point where increasing the number of clusters does not produce considerable improvement. When the number of clusters is 3, adding k makes no significant difference in the within-cluster sum of squares (WCSS). Consequently, the ITR and the PTR_{5mm} were clustered into 3 habitats. Based on the valuable features contained in Model 1, the wavelet-LLL_glcm_Imc2 features of Habitat-1, Habitat-2, and Habitat-3 of the ITR and the exponential_ngtdm_Busyness and wavelet-

LHL_glszm_SizeZoneNonUniformityNormalized features of Habitat-1, Habitat-2, and Habitat-3 of PTR_{5mm} were extracted. The results show that the exponential_ngtdm_Busyness of PTR_{5mm} Habitat-2 differed in PR expression (*P*=0.034) and Exponential_ngtdm_Busyness of PTR_{5mm} Habitat-3 differed differs in HER-2 expression (*P*=0.004). Habitat radiomics features with the distribution of ER, PR, HER-2, and Ki-67 expression are shown in the Appendix Table 3.

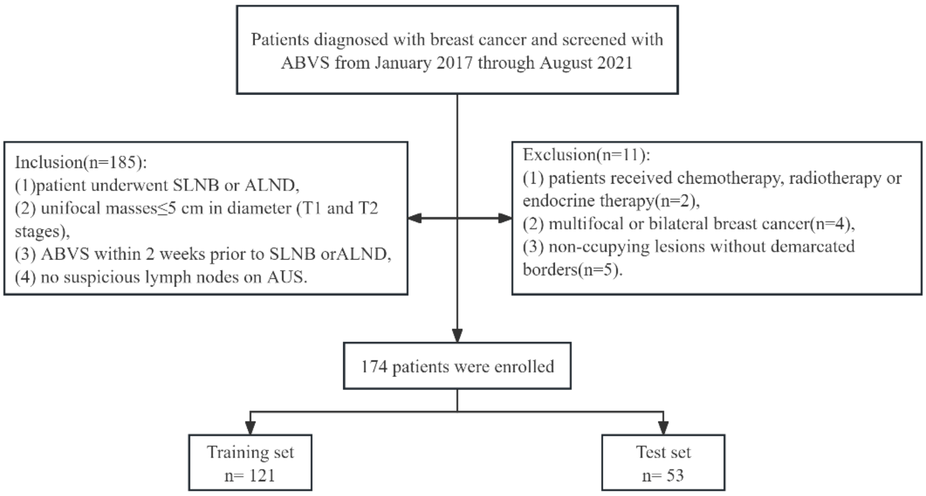

Appendix Fig.1. Flow chat of the patient recruitment.

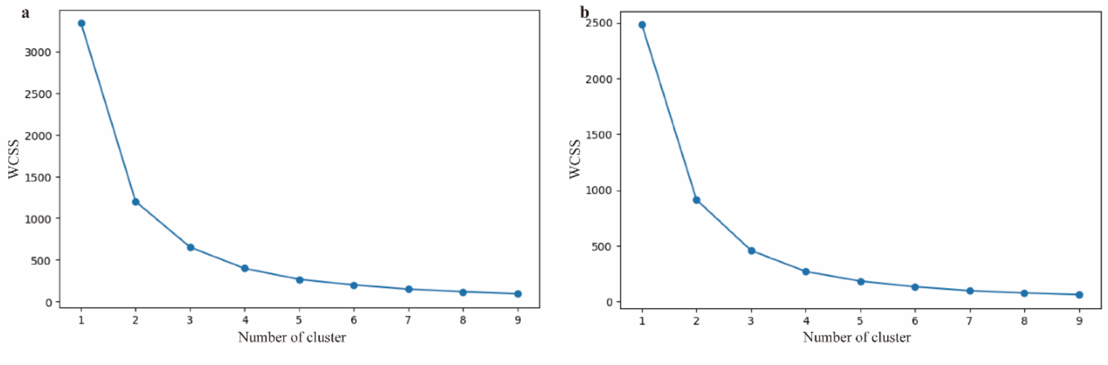

Appendix Fig.2. Intratumoral region (a) and Peritumoral 5 mm region (b) changes in WCSS with cluster number after feature extraction. When k = 3, increasing k doesn't produce a significant change in WCSS. WCSS, within-cluster sum of squares.

Appendix Table 1. The performance of models based on three machine learning classifiers.

Classifiers	Train					Test				
	AUC (95%CI)	ACC	SEN	SPE	*P values*	AUC (95%CI)	ACC	SEN	SPE	*P values*
**LR**	**0.718 (0.626-0.81)**	**0.661**	**0.814**	**0.577**	**Ref.**	**0.725 (0.560-0.889)**	**0.774**	**0.733**	**0.789**	**Ref.**
SVM	0.645 (0.574-0.715)	0.744	0.302	0.987	0.222	0.514 (0.412-0.616)	0.679	0.133	0.895	0.039
RF	0.565 (0.48-0.651)	0.628	0.349	0.782	0.001	0.701 (0.559-0.842)	0.774	0.533	0.868	0.783

P values were derived from the DeLong test. The boldfaced characters in the table represent the classifiers selected in this study. CI, confidence interval; LR, logistic regression; SVM, support vector machine; RF, random forest. Ref., reference; ACC, accuracy; SEN, sensitivity; SPE, specificity.

Appendix Table 2 The parameters of the intratumoral and peritumoral radiomics models

Models	Remaining features*	Remaining features#	Log(λ)	Feature for modeling LR	Coefficient
ITR	1335	139	0.123	wavelet.LLL_glcm_Imc2(ITR)	0.572
				wavelet.LLL_glszm_GrayLevelNonUniformityNormalized(ITR)	-0.464
ITR+ PTR_{1mm}	2776	309	0.149	original_gldm_LargeDependenceHighGrayLevelEmphasis(PTR_{1mm})	0.797
				wavelet.LLL_glcm_Imc2(ITR)	0.558
ITR+ PTR_{3mm}	2809	320	0.135	wavelet.LLL_glcm_Imc2 (ITR)	0.673
				exponential_ngtdm_Busyness(PTR_{3mm})	0.525
ITR+ PTR_{5mm}	2851	309	0.112	wavelet.LLL_glcm_Imc2 (ITR)	0.574
				exponential_ngtdm_Busyness(PTR_{5mm})	0.445
				wavelet.LHL_glszm_SizeZoneNonUniformityNormalized(PTR_{5mm})	-0.531
ITR+ PTR_{7mm}	2848	304	0.142	wavelet.LLL_glcm_Imc2 (ITR)	0.701
				exponential_ngtdm_Busyness(PTR_{7mm})	0.514
ITR+ PTR_{9mm}	2841	294	0.135	wavelet.LLL_glcm_Imc2 (ITR)	0.681
				exponential_ngtdm_Busyness(PTR_{9mm})	0.524

* Remaining features after ICCs, #Remaining features after correlation test. ICC, Intraclass correlation coefficients; ITR, intratumoral region; PTR, peritumoral region; LR, logistic regression.

Appendix Tables 3 Distribution of intra-tumoral and peritumoral habitat features in ER.PR, HER-2, Ki-67

Features		ER	*P*	PR	*P*	HER-2	*P*	Ki-67	*P*
Feature 1 (ITR Habitat1)	Negative	0.63±0.1	0.395	0.62±0.11	0.072	0.65±0.12	0.375	0.66±0.12	0.392
	Positive	0.65±0.12		0.65±0.11		0.63±0.11		0.64±0.11	
Feature 1 (ITR Habitat2)	Negative	0.67±0.1	0.277	0.68±0.11	0.639	0.68±0.1	0.967	0.68±0.12	0.805
	Positive	0.69±0.1		0.69±0.09		0.68±0.1		0.69±0.09	
Feature 1 (ITR Habitat3)	Negative	0.68(0.12)	0.87*	0.68±0.11	0.295	0.66±0.1	0.580	0.68±0.1	0.276
	Positive	0.67(0.17)		0.66±0.1		0.67±0.11		0.66±0.11	
Feature 2 (PTR_{5mm} Habitat1)	Negative	0(180.34)	0.302*	0(27.82)	0.902*	0(57.67)	0.200*	0(0)	0.18*
	Positive	0(0)		0(0)		0(0)		0(74.46)	
Feature 2 (PTR_{5mm} Habitat2)	Negative	0(147.78)	0.067*	0(144.68)	0.034*	69.14(288.79)	0.050*	0(173.21)	0.618*
	Positive	15.64(252.51)		27.66(274.16)		0(99.92)		0(241.85)	
Feature 2 (PTR_{5mm} Habitat3)	Negative	0(239.83)	0.439*	63.52(234.84)	0.164*	0(117.43)	0.004*	50.31(200.52)	0.45*
	Positive	0(174.74)		0(167.6)		121.81(285.97)		0(177.2)	
Feature 3 (PTR_{5mm} Habitat1)	Negative	0.24±0.03	0.393	0.25±0.03	0.893	0.25±0.04	0.507	0.24±0.03	0.641
	Positive	0.25±0.03		0.25±0.03		0.25±0.03		0.25±0.04	
Feature 3 (PTR_{5mm} Habitat2)	Negative	0.23(0.05)	0.474*	0.23(0.04)	0.132*	0.23(0.06)	0.104*	0.23(0.05)	0.889*
	Positive	0.23(0.05)		0.23(0.05)		0.24(0.04)		0.23(0.05)	
Feature 3 (PTR_{5mm} Habitat3)	Negative	0.23±0.04	0.32	0.23±0.04	0.131	0.24±0.04	0.088	0.23±0.04	0.12
	Positive	0.24±0.04		0.24±0.03		0.23±0.03		0.24±0.03	

* The Mann-Whitney U test was used. ER, estrogen receptor; PR, progesterone receptor; HER-2, human epidermal growth factor receptor 2; ITR, intratumor region; PTR, peritumoral region; Feature 1, wavelet-LLL_glcm_Imc2; Feature 2, exponential_ngtdm_Busyness; Feature 3, wavelet-LHL_glszm_SizeZoneNonUniformityNormalIzed.

Reference:

1. J. Shin, N. Seo, S. E. Baek, N. H. Son, J. S. Lim, N. K. Kim, W. S. Koom, S. Kim. MRI Radiomics Model Predicts Pathologic Complete Response of Rectal Cancer Following Chemoradiotherapy. Radiology. 2022;303(2):351-8.
2. Z. Fang, X. Yu, Q. Zeng. Random forest algorithm-based accurate prediction of chemical toxicity to Tetrahymena pyriformis. Toxicology. 2022;480:153325.
3. John C Platt. Fast training of support vector machines using sequential minimal optimization. Advances in kernel methods. 1999:185-208.
